# Understanding Integrated Community Case Management Institutionalization Processes Within National Health Systems in Malawi, Mali, and Rwanda: A Qualitative Study

**DOI:** 10.9745/GHSP-D-23-00509

**Published:** 2024-12-20

**Authors:** Alyssa L. Davis, Erica Felker-Kantor, Jehan Ahmed, Zachariah Jezman, Beh Kamate, John Munthali, Noella Umulisa, Oumar Yattara

**Affiliations:** a Consultant, U.S. President’s Malaria Initiative Impact Malaria, Washington, DC, USA.; b Population Services International, Washington, DC, USA.; c U.S. President’s Malaria Initiative Impact Malaria, Washington, DC, USA.; d U.S. President’s Malaria Initiative Impact Malaria, Lilongwe, Malawi.; e U.S. President’s Malaria Initiative Impact Malaria, Bamako, Mali.; f U.S. President’s Malaria Initiative Impact Malaria, Kigali, Rwanda.

## Abstract

**Introduction:**

Since 2012, the World Health Organization (WHO) and UNICEF have recommended integrated Community Case Management (iCCM) of childhood illnesses as an intervention delivered by community health workers (CHWs) in areas with limited access to health facilities to increase access to lifesaving interventions for children younger than 5 years with malaria, pneumonia, or diarrhea. In recent years, the importance of institutionalizing iCCM and community health more broadly within national health systems has become increasingly recognized.

**Methods:**

This qualitative study sought to identify and describe processes of iCCM institutionalization from the perspectives of health system actors. A total of 51 semistructured interviews were conducted with purposefully selected key informants in 3 countries: Malawi, Mali, and Rwanda. Thematic analysis of coded interview data was conducted, and country documentation was reviewed to provide contextual background for qualitative interpretation. The study was informed by a newly developed iCCM Institutionalization Framework, which conceptualizes the process of institutionalization through a maturity model of phases (i.e., awareness, experimentation, expansion, consolidation, and maturity) with 4 drivers: core values, leadership, resources, and policy.

**Results:**

According to key informant narrative descriptions, processes of iCCM institutionalization reflected a progression of maturity phases, which were iterative rather than linear in progression. All 4 drivers of institutionalization as conceptualized within the iCCM Institutionalization Framework were described by key informants as contributing to the advancement of iCCM institutionalization within their countries. Key informants emphasized the need to continually strengthen or reinforce iCCM institutionalization for it to be sustained within the context of wider health system dynamics.

**Conclusion:**

Overall, key informants viewed government ownership and integration within national systems to define the status of iCCM institutionalization. Further development of the iCCM Institutionalization Framework and other practical sensemaking models could assist health system actors in advancing institutionalization of iCCM and other health interventions.

## Abstract (precis)

Documenting and analyzing the processes of integrated community case management institutionalization across multiple country contexts can further understanding of institutionalization and development of practical sensemaking conceptual models.

## Teaser

**Key Findings:**

- To better understand the significant factors that affect the introduction, scale-up, and institutionalization of integrated community case management (iCCM) into national health systems in Malawi, Mali, and Rwanda, the perspectives of health system actors at various levels were explored and analyzed.
- Key informants across all 3 countries viewed government ownership and integration within national systems as defining the status of iCCM institutionalization.
- In each country, iCCM was described by key informants as being part of an overall wider country health agenda, which appeared to have supported a sense of ownership and advanced institutionalization within the countries.
- Key informants emphasized the need to secure sufficient financing and increases in domestic funds not only for iCCM services but the national health system as a whole.

**Key Implications:**

- Advocates for iCCM should consider positioning iCCM within wider country agendas to advance iCCM institutionalization within a national health system.
- Researchers should consider systematic document review, prospective analysis and complex adaptive systems approaches to further understanding of iCCM institutionalization processes and development of practical sensemaking models.

## INTRODUCTION

Integrated Community Case Management (iCCM) is a strategy to train, support, and supply community health workers (CHWs) to provide diagnostic, treatment, and referral services for children aged younger than 5 years for 3 common illnesses: malaria, pneumonia, and diarrhea.^1^ Since 2012, the World Health Organization (WHO) and UNICEF have recommended iCCM as a key equity-focused global health strategy delivered by CHWs to increase access to lifesaving interventions for children aged younger than 5 years in areas with limited access to health facilities.^2^^,^^3^ iCCM has been widely adopted, particularly in sub-Saharan Africa, where more than 30 countries have reported iCCM policies and implementation of services by CHWs.^4^ Evidence from these programs demonstrates that appropriately trained, supervised, and supplied CHWs are able to correctly identify, classify, and treat most children who have uncomplicated cases of these illnesses and can refer those with danger signs to health facilities, helping to mitigate severe illness and mortality.^5^ A review of 8 studies in 6 sub-Saharan Africa countries (Cameroon, Ethiopia, Ghana, Sierra Leone, Uganda, and Zambia) reported consistent declines in child mortality (ages 2–59 months) in geographic areas with iCCM programs compared to control areas.^6^ An assessment of iCCM programs in 5 sub-Saharan Africa (Niger, Nigeria, DRC, Malawi, and Mozambique) countries, for example, estimated an average decline in mortality in children aged younger than 5 years of 9.9% in areas with iCCM services.^7^ Finally, a review of Demographic Health Survey data across 21 countries over the period 2010–2018 concluded there is evidence that CHWs providing iCCM services have contributed to reducing inequities in health care coverage by decreasing treatment delays and targeting underserved populations.^8^

Despite widespread adoption of iCCM across Africa, only a small number of countries have been successful in fully translating policy to implementation, resulting in national iCCM programs covering most or all targeted areas.^9^ Many reasons have been documented as sources of difficulties that countries have faced in scaling up, including a lack of supportive policy change; inadequate financing, human resources, and supply chains; and poor coordination across programs involved in iCCM.^10^^–^^12^ Given these challenges, the importance of institutionalizing iCCM and community health more broadly within national health systems has become increasingly recognized.^13^^,^^14^ However, this recognition has been in the absence of an explicit definition for iCCM institutionalization and without clarity on the distinctions or relationships between other concepts of interest to public health practitioners, such as intervention fidelity, scale, and sustainability.

Two key documents have provided insight into the conceptualization of iCCM institutionalization to date. One is a scoping review of policy documents and publications to identify models of and gaps in the institutionalization of iCCM benchmark components into national health systems with the aim of drawing lessons for future iCCM implementation and sustainability.^15^ The other is a technical consultation report that presents a range of recommendations to advance the institutionalization of iCCM, which were agreed upon by technical experts convened by UNICEF and the WHO at a technical consultation in 2019.^13^ These documents provide important insight into how iCCM institutionalization has been conceptualized by relevant technical experts but with notable limitations. First, while both documents emphasize the importance of integration into national health systems and principles of health system strengthening, neither puts forward an explicit definition of institutionalization in relation to iCCM. Second, both documents use the iCCM benchmark components originally developed by McGorman et al. in 2012 as an organizing framework, but neither document addresses how institutionalization happens (i.e., processes of institutionalization) within or across the benchmark components.^16^ The scoping review uses the benchmark components (i.e., coordination and policy setting, costing and financing, human resources, supply chain management, service delivery and referral, communication and social mobilization, supervision and performance quality assurance, and monitoring, evaluation and health information systems) to analyze approaches to institutionalization across countries, while the technical consultation report uses the components to categorize recommendations.^16^

Given the limited documentation of what institutionalization of iCCM means and how institutionalization of iCCM occurs within a particular health system context, we conducted a qualitative study aimed at documenting and understanding the processes of iCCM institutionalization from the perspective of health system actors in 3 countries: Malawi, Mali, and Rwanda. The study was informed by a new conceptual framework for iCCM institutionalization developed as part of a collaboration between the President’s Malaria Initiative (PMI) Impact Malaria project and the Child Health Task Force to create an iCCM institutionalization toolkit.^16^^,^^17^ The framework is intended to be a simple sensemaking tool to assist national health system actors in prioritizing their efforts to advance iCCM institutionalization. The framework defines institutionalization as “a process and end state by which iCCM becomes an integral, routine and stable part of both community and health systems,” thereby conceptualizing institutionalization as both a process and an end state of stability, which should not be viewed as a perpetual fixed state, but one that can still change through a new process of institutionalization over time.^17^ This definition and framework are informed by institutional theory as well as efforts to define and assess institutionalization within the context of health systems.^18^^–^^22^

**FIGURE 1:**
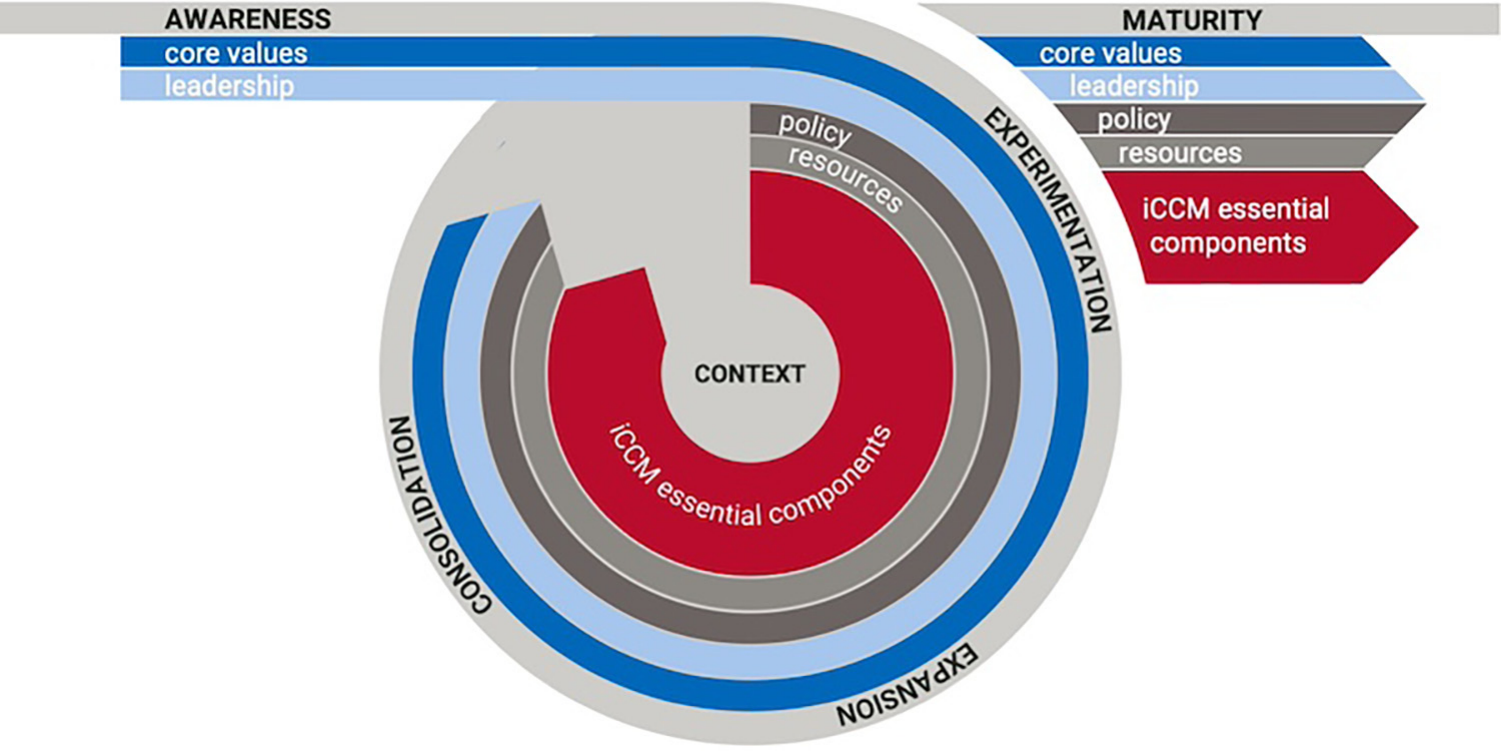
Integrated Community Case Management Institutionalization Framework^13^

Figure 1 illustrates the process of institutionalization through a maturity model of phases (i.e., awareness, experimentation, expansion, consolidation, and maturity) with 4 drivers: core values, leadership, resources, and policy. Context is depicted at the center to reflect that the process of institutionalization starts with and must be responsive to dynamics within the country context (e.g., cultural values, health service delivery structures, and political systems). It includes the iCCM Essential Components (i.e., the benchmark components originally defined by McGorman et al.) to reflect the importance of maintaining all essential implementation elements throughout the process of institutionalization. Within this framework, changes across the drivers are thought to enable transition through the maturity phases, which may not progress linearly but actually regress, stagnate, or oscillate, shown as a loop in the figure. More background on the literature review, theoretical underpinnings, and development process of this framework can be found elsewhere.^16^^,^^17^

This qualitative study intended to use the iCCM Institutionalization Framework to broadly and flexibly frame the exploration of the concept and processes of iCCM institutionalization with national health system actors, not evaluate the status of iCCM institutionalization within a country. The study intended to document the unique narrative descriptions of if, how, and why iCCM had been institutionalized according to the perspectives of health system actors within each country. Documenting and analyzing these narratives can help identify common patterns across multiple contexts and inform global understandings of iCCM institutionalization from a more grounded national health system perspective, which can then be applied to further develop and refine the iCCM Institutionalization Framework and other practical, sensemaking models intended to support actors at the forefront of advancing iCCM institutionalization within their national health systems.

The study intended to document the unique narrative descriptions of if, how, and why iCCM had been institutionalized.

The study aimed to (1) document through narrative descriptions the progression of introduction, scale-up, and institutionalization of iCCM; (2) identify if and how maturity phases of the iCCM Institutionalization Framework were reflected in these narrative descriptions; and (3) describe if and how each driver of iCCM Institutionalization Framework (i.e., core values, leadership, policy, and resources) contributed to iCCM institutionalization from the perspectives of national health system actors.

## METHODS

This study employed a qualitative research approach, which centered on primary data collection through semistructured interviews with a range of purposefully selected key informants in the 3 selected study countries. The interviews were supplemented by a review of secondary documents (e.g., national health policy documents, national health plans, child health strategies, and other documents related to iCCM implementation in the country), including documents referred to by key informants, to provide contextual background for the interpretation of qualitative interview data.

### Country Selection

Three countries identified as implementing iCCM at national scale and advancing toward institutionalization were selected for inclusion in this study: Malawi, Mali, and Rwanda. Two key sources of evidence informed this selection: (1) a 2018 thematic review report that identified 7 sub-Saharan Africa countries as implementing iCCM at national scale (i.e., Cameroon, Ethiopia, Malawi, Mali, Rwanda, Senegal, and Zambia);^12^ and (2) a scoping review by Nanyonjo et al. in 2019 that compared best practice compliance with iCCM benchmark components by low- to middle-income countries.^15^ According to the scoping review, Malawi demonstrated best practice compliance with the highest number of benchmark components (i.e., 6 of 8), followed by Ghana, Ethiopia, and Rwanda (i.e., 5 of 8), while all other countries complied with a lower number of the benchmark components, including Mali (i.e., 1 of 8). Based on review of this information, Malawi, Mali, and Rwanda were selected to provide a combination of country contexts in terms of the extent of compliance with benchmark components, which could be viewed as a proxy measure for the extent of iCCM institutionalization; variety of CHW models (e.g., salaried vs. volunteer); and geography (i.e., Southern Africa, West Africa, and East Africa). Finally, preference was given to countries within the portfolio of PMI’s flagship global service delivery project, Impact Malaria, which supported this study.

Table 1 provides an overview of key indicators related to the status of child health and care-seeking for key childhood illnesses over the timeframe considered by the study (i.e., the last 20 years before interviews for the study were conducted in early 2023). This table draws on data from national Demographic Health Surveys and Multiple Indicator Cluster Surveys conducted in each country; the most recent surveys were conducted in 2023–2024 for Mali and in 2019–2020 for Malawi and Rwanda.^23^^,^^24^ Across all 3 countries, care-seeking for key childhood illnesses increased between 2004 and 2018. However, in the most recent years of measurement, only Rwanda has maintained this upward trajectory, while Malawi and Mali have shown some declines in care-seeking. Between 2004 and 2024, mortality rates for children aged younger than 5 years fell substantially and consistently across all 3 countries. This qualitative study did not aim to assess the contributions of iCCM to increases in care-seeking for or treatment of childhood illnesses nor decreases in mortality; these figures are provided solely as background for interpreting key informant narratives on iCCM institutionalization and child survival progress within the 3 selected countries. Additionally, Table 2 provides an overview of the iCCM service delivery arrangements when the study commenced in late 2022. It should be noted that iCCM service packages and service delivery arrangements within each of the 3 countries evolved across the timeframe considered by the study; this evolution is a part of the key informant’s narrative descriptions of the introduction, scale-up, and institutionalization of iCCM within their country contexts.

**TABLE 1.** Illness Care-Seeking, Treatment, and Mortality Indicators for Children Aged Younger Than Five Years, Malawi, Mali, and Rwanda^a^

	**Children Aged Younger Than 5 Years**				
	**With Diarrhea for Whom Treatment/Advice Was Sought**^b^ **(Sought From CHW), %**	**With Fever for Whom Treatment/Advice Sought**^b^ **(Sought From CHW), %**	**With Fever Who Received Antimalarials, %**	**With ARI Symptoms for Whom Treatment/Advice Sought**^b^ **(Sought From CHW), %**	**Mortality Rate, per 1,000 live births**
**Malawi** ^c^					
2004	36.4 (2.8)	15.9 (0.5)	28.4	36.5 (0.8)	133
2010	62.1 (3.3)	64.6 (1.8)	43.4	70.3 (2.2)	127
2015–2016	65.8 (4.4)	66.9 (3.4)	38.9	77.6 (4.3)	63
2019-2020	56.9 (6.8)^d^	62.6 (6.0)^d^	30.7^d^	70.4 (4.1)^d^	56^d^
**Mali**					
2006	17.8 (NA)	32.8 (NA)	31.7	38.1 (NA)	191
2012–2013	31.8 (1.3)	32.0 (0.8)	22.5	31.2 (0)	95
2018	49.0 (2.5)	52.8 (2.8)	31.0	70.9 (5.6)	101
2023–2024^e^	37.1 (NA)	50.6 (NA)	45.3 (NA)	47.4 (NA)	87
**Rwanda**					
2005	14.1 (0.43)	28.5 (0.6)	12.3	27.9 (0.5)	152
2010	37.2 (12.9)	42.7 (15.6)	10.8	50.2 (13.0)	76
2014–2015	43.6 (10.4)	56.7 (12.6)	11.4	53.9 (14.2)	50
2019–2020	51.9 (7.5)	62.3 (10.6)	NA	72.7 (11.8)	45

Abbreviations: ARI, acute respiratory infection; CHW, community health worker; NA, not available.

a All data from Demographic and Health Surveys for respective countries and years noted, unless otherwise indicated.

b Treatment/advice sought from a health facility or provider, including CHWs.

c Figures from Malawi DHS datasets for treatment sought from CHWs include responses coded as public or private sector, CHW or health surveillance assistant.

d Data from Malawi Multiple Indicator Cluster Survey Report.^24^ Note: Community health providers include both public (community health worker and mobile/outreach clinic) and private (nongovernment community health worker and mobile clinic).

e Dataset not yet available, so available indicators reported from Mali Demographic and Health Survey 2023–2024 Key Indicators Report.^23^

**TABLE 2.** Overview of iCCM Service Delivery Arrangements in Malawi, Mali, and Rwanda, 2022

	**Malawi**	**Mali**	**Rwanda**
CHW providing services	Health surveillance assistant	Agents de santé communautaire	Agents de santé binôme
Full time or part time	Full time	Full time	Part time
Employed or volunteer	Employed	Employed	Volunteer
Pre-service training length	∼12 weeks	∼22 days	∼12 weeks
Salary or incentive amount^a^	∼175–280/month	∼ 70/month	Variable amounts (performance-based incentives paid to cooperatives, tasked to allocate 70% toward income-generating activities and 30% towards payments to members)
Supervisor	Senior health surveillance assistants	Dedicated supervisor from the Centre de Santé Communautaire (community health center)	Cell coordinators
CHW to population ratio	1/1,000 people	1/100–700 people	2/100–150 households
Facility or community based	Facility with outreach to multiple communities	Community	Community (2 per village)
iCCM services	Include management of uncomplicated cases of malaria, pneumonia, diarrhea, newborn sepsis, malnutrition, and conjunctivitis, as well as severe cases to the nearest health facility.	Part of essential care in the community service package, which includes case management of diarrhea, cough (pneumonia), malaria and malnutrition for children aged younger than 5 years, family planning, HIV/TB case management, malaria case management for pregnant women, water sanitation and hygiene surveillance, as well as health center referrals.	Include assessment, classification, and treatment or referral of diarrhea, pneumonia, malaria, and malnutrition in children aged younger than 5 years; diagnosis and treatment of malaria for those aged older than 5 years.
Targeting of iCCM services	Health surveillance assistants schedule “village clinics” in hard-to-reach communities (i.e., >8 km from a health facility and/or other geographical barriers) to provide iCCM services.	Agents de santé communautaires in communities >5 km from a community health center and areas geographically difficult to access provide iCCM services.	Agents de santé binôme in all villages provide iCCM services.

Abbreviations: CHW, community health worker; iCCM, integrated community case management.

a Equivalent amounts calculated as of June 2023.

### Key Informant Selection

Key informants were initially identified based on the national health sector knowledge of country-based PMI Impact Malaria project staff and review of available country documentation (i.e., country policy, program, and research documentation). Purposeful sampling was guided by the principle of identifying individuals with a variety of vantage points on the introduction, scale-up, and institutionalization of iCCM in each country through direct country-based experience over the past 5–10 years. Key informants included officials of the Ministry of Health (MOH); representatives of donor, technical, and implementation partner organizations; representatives from wider civil society agencies engaged in community health; CHWs; and community leaders. Key informants were primarily country nationals with extensive insight into the national government and local context; many had held multiple positions across government, donor and other health sector partner roles over the course of their careers and engagement with iCCM. Key informant selection allowed for referrals where initial key informants could recommend individuals who could provide additional insights relevant to the study. At a local level, CHWs were purposefully selected from health districts where the PMI Impact Malaria project was engaged programmatically in each country. This was done in consultation with respective MOH representatives at the local level to identify long-serving CHWs (i.e., those working within their role for at least 5 years) with a roughly equal balance of men and women. In Mali, both CHWs and community leaders were interviewed to enable gender balance of key informants at the community level. A total of 51 key informants were interviewed, including 17 key informants in Malawi, 15 in Mali, and 19 in Rwanda (Table 3).

**TABLE 3.** Overview of Key Informants Interviewed

	**Malawi**	**Mali**	**Rwanda**	**Total**
Civil society/ nongovernmental organizations	0	4	0	4
Community health workers	8	4	10	22
Men	5	0	6	
Women	3	4	4	
Community leaders	0	4	0	4
Government	6	3	5	14
United Nations agencies	3	0	4	7
Total	17	15	19	51

### Data Collection

Interviews with key informants from national and subnational levels were conducted over Zoom or phone in English or French. Interviews with key informants at the local level, including CHWs and community leaders, were conducted in person in relevant local languages (i.e., Bambara in Mali, Chichewa in Malawi, and Kinyarwanda in Rwanda). All interviews were recorded with the permission of the respondent after the respondents gave their informed consent.

Interviews were conducted between January and April 2023 using a semistructured interview guide to explore the following: (1) key informant’s engagement with iCCM, both length of time and roles; (2) the country’s journey of introducing, scaling up, and institutionalizing iCCM; (3) current status of iCCM institutionalization; (4) any influence of iCCM institutionalization drivers (i.e., core values, leadership, policy, and resourcing) in advancing institutionalization; (5) challenges encountered in advancing iCCM institutionalization; (6) strategies used to overcome challenges; and (7) lessons learned from the country’s iCCM institutionalization journey (Supplement 1).

Country documentation (e.g., national health policy documents, national health plans, child health strategies, and other documents related to iCCM implementation in the country) was gathered and reviewed to inform and provide context for analysis of the qualitative interview data. Relevant documents were identified before conducting interviews as well as throughout the interview process, based on documents referred to and, in some cases, shared by key informants.

### Data Analysis

All interviews conducted virtually in English or French were transcribed using Amberscript software and then reviewed and corrected for accuracy by the interviewer. Interviews conducted in person in local languages with CHWs and community leaders were transcribed manually and translated into English or French by the interviewer. All interview transcripts were uploaded to Dedoose software for data management and thematic analysis. A codebook of deductive codes was developed based on the conceptual framework and study objectives (Supplement 2). One research team member coded each transcript, and then all transcripts and coded excerpts were reviewed by the 2 research team members responsible for conducting the data analysis. Based on the review of coded data, observations and patterns were identified across data from each country and then compared for similarities and differences across the 3 countries. Research team focal points in each country provided contextual background and clarifications for the interpretation of findings.

### Ethical Approval

Ethics review and approval were obtained from the respective ethics review bodies in each country: Comité National d’Éthique pour la Santé et les Sciences de la Vie, Ministère de la Santé et du Développement Social, Mali (N°2022159/MSDS-CNESS); National Health Sciences Research Committee, Ministry of Health, Malawi (#22/08/2960); and National Health Research Committee, Ministry of Health, Rwanda (NHRC/2022/PROT/045). The Population Service International’s Research Ethics Board gave the study a determination of “non-human subjects research.”

## RESULTS

Key informants described their country’s journey from initial introduction of iCCM to its current status of institutionalization. Based on their responses, a narrative description of each country’s journey was constructed, including key evidence and documentation referenced by key informants.

### Malawi’s Integrated Community Case Management Institutionalization Journey

#### Overview

Malawi adopted iCCM in 2008 using government-employed health surveillance assistants (HSAs) to deliver essential services in hard-to-reach areas, where access to facility-based health services was inhibited by distance (more than 8 km) and other geographical features (e.g., rivers and lack of roads). The HSAs conduct “village clinics” (i.e., scheduled events held in the community) where they manage uncomplicated cases of malaria, pneumonia, diarrhea, newborn sepsis, malnutrition, and conjunctivitis and refer severe cases to the nearest health facility. Following the initial pilot in 2008, iCCM was scaled up with the support of external partners to train and supply HSAs to deliver services across expanding geographical areas. The process of developing and rolling out Malawi’s first Community Health Strategy (2017–2022) was an important part of progressing iCCM institutionalization through strong government leadership and improved coordination across partners. To date, iCCM is implemented in hard-to-reach areas of all 29 districts of the country.

#### Timeline

**FIGURE 2:**
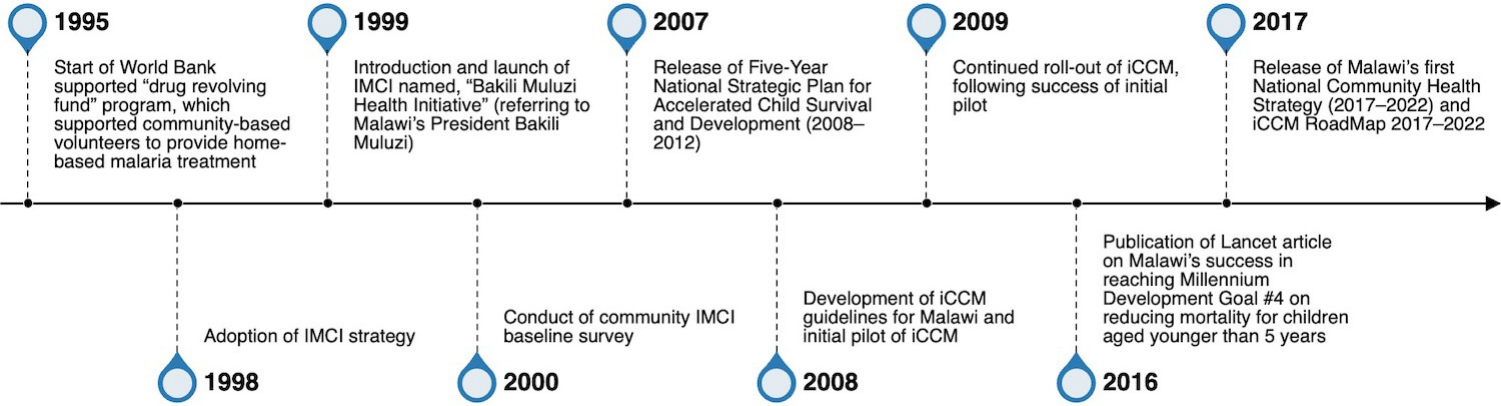
Key Events in Malawi’s iCCM Institutionalization Process Abbreviations: iCCM, integrated community case management; IMCI, integrated management of childhood illness.

Key informants described iCCM institutionalization as a gradual process with key events in this process spanning from 1995 to 2017 (Figure 2). Some key informants described the iCCM approach as an extension of the country’s integrated management of childhood illness (IMCI) approach, which was initially adopted in 1998, and as a key feature of the country’s community health program.

#### Status of iCCM Institutionalization

All national-level informants described iCCM as being well institutionalized or even “fully” institutionalized in Malawi. The rationales stated for this were most frequently that it was a government-led initiative that had a dedicated unit (i.e., the IMCI Unit) and was implemented by paid government staff (i.e., HSAs) at the community level. Key informants at the national level also noted how iCCM was enshrined within Malawi’s national health policies and was a core feature of the country’s community health program. However, views on the status of iCCM institutionalization were more mixed at subnational and community levels, with some informants indicating that it was not fully institutionalized, as not all HSAs provide iCCM services (given the national strategy of targeting areas considered hard to reach). Several HSA key informants felt that all HSAs should receive training and supplies to provide iCCM services for iCCM to be fully institutionalized in the country.

All national-level informants described iCCM as being well institutionalized or even “fully” institutionalized in Malawi.

*I feel it is [institutionalized] because it is not taken as a vertical intervention as of now. Now it is a critical part, as an intervention that government sees or looks at as essential to advance or to support the primary health care work on the ground. … Implementation of iCCM in Malawi is done by the formal primary health care providers, which are HSAs and these are a paid up cadre by government. So, it’s not like a project. A project could just support probably capacity-building or something like that. … It is the lowest cadre of government that provide this [iCCM services].* —United Nations (UN) key informant, Malawi

*iCCM program should involve all HSAs, not only those in hard-reach areas, for iCCM [institutionalization] to improve.* —CHW key informant, Malawi

### Mali’s Integrated Community Case Management Institutionalization Journey

#### Overview

Since 2010, Mali has implemented iCCM as a part of a nationally defined package of community health services, soins essentiel dans la communauté (SEC), which can be translated as “essential care in the community.” The SEC package includes case management of cough (pneumonia), diarrhea, malaria, and malnutrition for children aged younger than 5 years; family planning; HIV/TB case management; malaria case management for pregnant women; water, sanitation, and hygiene surveillance; and health center referrals. Key informants described 3 major phases of SEC institutionalization, which align with phases of SEC strategy development described in the National SEC Strategic Plan 2021–2025. The first phase (2010–2015) involved the initial development of the SEC strategy, implementation with external donor support, and establishment of coordination mechanisms (i.e., national and regional steering committees). The second phase (2016–2020) involved the refinement of the strategy, expansion of geographic coverage, and increase in service package scope (e.g., addition of HIV and TB interventions), as well as greater health system integration (i.e., national health information, supply chains, and supervision systems). The third phase (2021–present) has involved the adoption of a legal decree (2022) that formalized the status of CHWs, known locally as agents de santé communautaire (ASCs), as recognized full-time and paid health workers within the government health system, along with work to develop a sustainable financing plan and strengthen management mechanisms, including digitization of the community health information system and development of an electronic payment system. To date, the SEC package has been implemented across all regions of Mali by ASCs more than 5 km from a health facility with limited coverage, particularly in the north due to insecurity and political instability.^25^

#### Timeline

**FIGURE 3:**
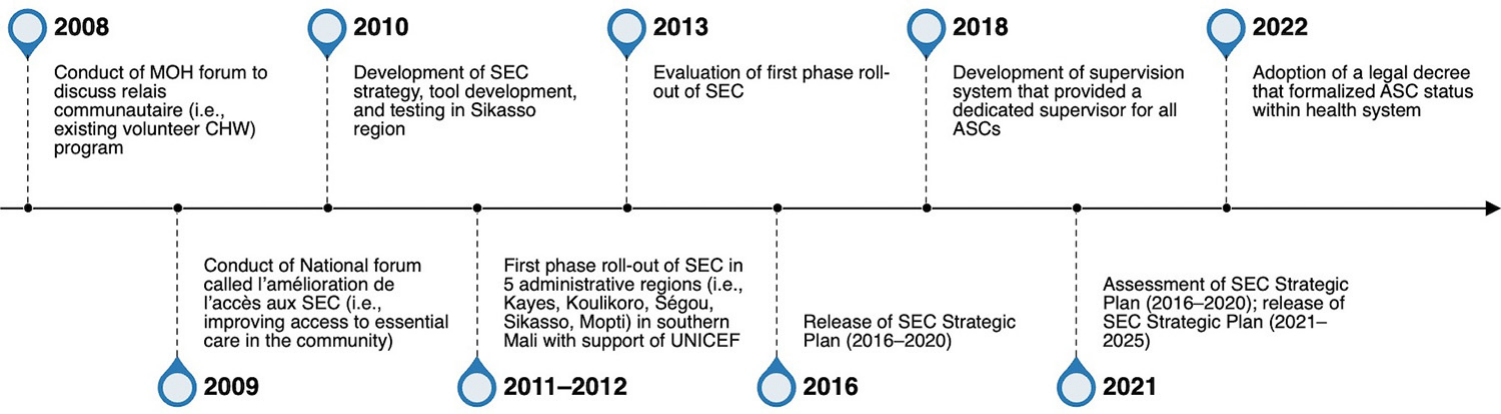
Key Events in Mali’s iCCM Institutionalization Process Abbreviations: ASC, agents de santé communautaire; CHW, community health worker; iCCM, integrated community case management; MOH, Ministry of Health; SEC; soins essentiel dans la communauté.

Key informants did not distinguish between iCCM and the country’s SEC package; rather, they described iCCM as being a part of the development, scale-up, and institutionalization of the SEC package, with key events in the process spanning from 2008 to 2022 (Figure 3).

#### Current Status of iCCM Institutionalization

Across key informants, there was consensus that the SEC package was institutionalized in Mali. The main evidence cited for this was that in 2022, the government signed a decree, which is the legal process used in Mali to formally recognize a government policy or program.^26^ The main challenge noted by key informants at present was the need to have SEC funded by the government through a line item in the budget. The community health working group chaired by the subdirectorate in charge of community health under the Director General of Health is advocating for government funding for ASC salaries, which are currently funded by donors. According to key informants, SEC is a national strategy that has been implemented across all regions in Mali, although lack of access due to violence and insecurity has resulted in less than full coverage in some regions. The level of coverage of services across the country did not factor into key informant viewpoints on the status of iCCM institutionalization.

SEC is a national strategy that has been implemented across all regions in Mali.

*I think that the SEC strategy is now institutionalized in the health system. The ASCs are recognized by the system. They are an integral part of the health system, starting at the peripheral level, which is the Community Health Center. So now we have a text that recognizes that the ASC is attached to the Community Health Center.* —Nongovernmental organization (NGO) key informant, Mali

*I say it’s institutionalized now with the signing of the decree, it’s a presidential decree. —*Government key informant, Mali

### Rwanda’s Integrated Community Case Management Institutionalization Journey

#### Overview

Since 2009, Rwanda has implemented the iCCM approach, starting as a pilot in 6 health districts and expanding quickly in 2010–2011 to all 30 health districts. During this rapid expansion, every village in Rwanda elected 2 community members (1 man and 1 woman) to become agents de santé binômes (paired health workers), usually referred to simply as binômes. By 2013, every village had 1 binôme (female and male CHW pair) in charge of iCCM services and an animatrice de santé maternelle in charge of maternal and newborn health. Key informants described iCCM institutionalization as a process that originated with Rwanda’s community health program in 1995 following the 1994 genocide in Rwanda, although case management of malaria was not introduced until 2004 and iCCM in 2009. Successive iterations of government-led evaluation, strategic planning, and policy dialogue were described as instrumental in progressing iCCM institutionalization as a part of the national community health program, particularly the Rwanda Biomedical Center-commissioned evaluation of the community health program in 2016, development of a National Community Health Strategic Plan in 2017, and the national policy dialogue held in 2022. The national policy dialogue resulted in an MOH decision to transition to a new “polyvalent” (i.e., multitask) CHW system where all CHWs, including the existing binômes and animatrices de santé maternelle, would provide the same integrated service package across all areas of the country.

#### Timeline

**FIGURE 4:**
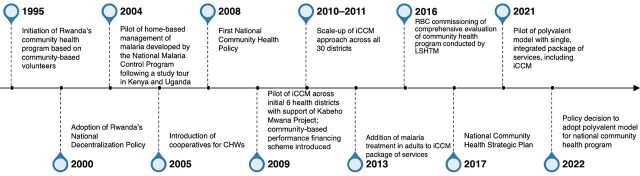
Key Events in Rwanda’s iCCM Institutionalization Process Abbreviations: CHW, community health worker; iCCM, integrated community case management; IMCI, integrated management of childhood illness; LSHTM, London School of Hygiene & Tropical Medicine; RBC, Rwanda Biomedical Center.

Most key informants saw iCCM institutionalization as a part of a wider process of institutionalizing community health within Rwanda’s health system, with key events in this process spanning from 1995 to 2022 (Figure 4).

#### Status of iCCM Institutionalization

There was strong consensus across key informants that iCCM is fully institutionalized within the Rwandan health system. Key informants highlighted that the iCCM approach (and broader community health approach) is owned by the government, implemented by government institutions (i.e., not dependent on partner mechanisms), and fully embedded within existing health systems (e.g., supply chains, health information, and financing strategy). Specific examples cited as evidence of iCCM institutionalization included: iCCM was scaled up nationally and reached all populations in need, including those who were poor and hard to reach; iCCM reporting went through national reporting systems and was integrated into the national health information system; the government issued laws, policies, and strategies to govern all aspects of the community health program, including delivery of iCCM services.

There was strong consensus across key informants that iCCM is fully institutionalized within the Rwandan health system.

*I would say that really the ownership is high. Of course, we have external partners on board supporting financially, technically, but they are all coordinated and the ownership is on the government side*. —Government key informant, Rwanda

*[Institutionalized] 100%, because it is implemented nationwide, monitored, and supplied through government systems.* —UN key informant, Rwanda

*We treat [more] cases than the health center. It is no doubt one of the health system levels in Rwanda.* —CHW key informant, Rwanda

### Progression of Institutionalization Across Maturity Phases

Key informants’ narrative descriptions of their country’s iCCM institutionalization journey were analyzed to see if and how maturity phases of the iCCM Institutionalization Framework were reflected and examined for similarities and differences across the 3 countries.

#### Awareness

Across all 3 countries, key informants described initial interest in iCCM as driven by a lack of access to primary health care and trends in morbidity and mortality at the community level (e.g., low and delayed care-seeking and high rates of mortality in communities with less access to health facilities). Key informants in all 3 countries described the introduction of iCCM as building on previous experiences with home-based management of malaria, specifically protocols involving presumptive treatment of fever (before the widespread availability of rapid diagnostic tests for malaria) by volunteer CHWs (i.e., malaria volunteers in Malawi; relais communautaires in Mali; binômes in Rwanda). Both UNICEF and WHO were commonly referred to as having a role in developing iCCM and supporting its initiation in each country. However, there were differences across the 3 countries in terms of how key informants perceived the introduction of iCCM. In Malawi, key informants described the beginning of iCCM as a more discrete temporal event (i.e., introduced as a specific, named initiative). Key informants in Rwanda and Mali described iCCM more generally as emerging as a part of their national community health programs. Furthermore, key informants in Mali made no distinction between iCCM and the country’s SEC package, so it was difficult to probe the origins and evolution of iCCM specifically. However, this, in itself, is a finding that points to iCCM being considered an inextricable component of the country’s SEC package.

*The gaps [lack of health care access] were realized somewhere around 2007, but the actual interventions on the ground started in around third quarter of 2008. … That was actually when the first community health workers were trained and put on the ground. And then since that last third quarter of 2008, until now, government partners, everybody has seen the importance of this. I’ve seen the contribution it has made towards the reduction of child mortality and it has remained positive too, since that time until today.* —NGO key informant, Malawi

*The history of the community health system is back, I don’t recall exactly if it is 2000 or before that when the MOH conducted the assessment and found out that many people were getting sick in the community and not accessing services.* —UN key informant, Rwanda

*It was decided to bring care closer to the household level, not the communes, but right down to the household level for health care, and this is when essential community care [SEC package] was adopted as a strategy that is scientifically and socially valid.* —NGO key informant, Mali

#### Experimentation

Key informants across all 3 countries discussed an initial iCCM (or SEC package in Mali) pilot involving the development and testing of training materials and tools within a small number of selected health districts. The mention of donor, technical, or implementation partners being involved in this process was common. These initial pilots occurred over relatively short periods of time (i.e., around 6 months to 1 year). In Malawi and Rwanda, pilots of home-based management of malaria were noted to have happened before pilots of iCCM. Across all 3 countries, key informants described these pilots as being clearly successful, both in terms of demonstrating the feasibility of the model as well as noting evidence of decreases in care-seeking timeframes and increases in treatment coverage. Sources of evidence were described as coming from formal assessments and routine data, as well as feedback from community members.

Key informants across all 3 countries discussed an initial pilot involving the development and testing of training materials and tools within a small number of selected health districts.

*In 2009 and 2010, the tools were developed, and SEC was piloted in the region of Sikasso in 2011. Due to the success of SEC in Sikasso, the approach was implemented in Kayes, Koulikoro, Ségou, and Mopti later that year*. —NGO key informant, Mali

*After the pre-testing, we finalized the tools and our first introduction of iCCM was in October in the same year, 2008. So, you notice that from June to October, that was a very short period of time. But since then, we started working in 10 districts. These 10 districts are the ones that were like the high burden in terms of under-5 mortality in Malawi, looking at the DHS reports.* —MOH key informant, Malawi

*Rwanda has started iCCM since 2003–2005, it was piloting [home-based management of malaria] and this started in 3 districts to see how this can be done and after 1 year of implementing. So, it shows that it was something which is feasible.* —Government key informant, Rwanda

In addition to initial pilots of iCCM, key informants noted iterative processes of experimentation related to testing adaptations of treatment protocols (e.g., use of rapid diagnostic tests and rectal artesunate), service packages (e.g., treatment of malaria in adults and management of malnutrition in children) and other improvements in data collection, supervision, and other operational considerations (e.g., types and roles of CHWs and their relationship to the health system). These experimentation efforts were described as taking place both during and after scale-up of iCCM, indicating that phases of experimentation and expansion were not necessarily distinct but may have occurred simultaneously. Additionally, key informant narratives suggested that experimentation remained an important feature throughout their countries’ institutionalization journeys. Processes of experimentation were also described as interlinked with efforts to improve program functionality and integration with the health system.

#### Expansion

Across all 3 countries, key informants described a rapid decision to scale up nationwide after the initial iCCM pilot, although how this was defined and how fully it was achieved varied. In Rwanda, key informants described nationwide scale as the selection and training of binômes across all communities in the country. In Mali, key informants referenced the SEC strategy as being at nationwide scale, although implementation had not expanded to some areas of the country experiencing conflict, and, as such, nationwide scale was explained in terms of national policy and intention to expand implementation to all areas. In Malawi, key informants usually described nationwide scale as applying a national strategy of targeting hard-to-reach communities across all health districts in the country, although some key informants, particularly HSAs themselves, noted their view that all communities should be targeted and iCCM could only be considered fully institutionalized if this were the case.

*Increasing treatment was one of the main objectives of establishing iCCM, you know, with hard-to-reach communities that don’t have access or very limited access with child health care services. So that was one of the prerequisites when we started moving towards the direction of scaling up.* —MOH key informant, Malawi

*I remember the scale-up happened around 2011–2012. I was part of the scaling up to reach in every district in every village in rural Rwanda.* —UN key informant, Rwanda

Overall, key informants did not describe in detail particular scale-up plans or strategies but generally expressed the intention to scale nationwide (as defined or understood within the country context) after successful pilots. While expansion focused on increasing geographical coverage, this was predicated on increases in the total number of CHWs providing iCCM services, either through recruiting new CHWs or training existing CHWs. In Rwanda, expansion of iCCM services was accompanied by a nationwide scale-up of the election of binômes by their communities. In Mali, implementation of the SEC strategy involved a rapid selection and introduction of ASCs to new communities. In Malawi, expansion included training of existing HSAs on iCCM and waves of new recruitment of HSAs, sometimes supported by international donors. Therefore, key informant descriptions of the expansion of iCCM implementation were inherently linked with processes of recruiting and deploying CHWs to deliver iCCM services.

#### Consolidation

After initial pilots and efforts to scale up iCCM services, key informants described efforts to improve the functionality and integration of operational arrangements to deliver iCCM services within the health system. These efforts were often described as linked with iterative processes of experimentation. Key informants across all 3 countries described changes related to health information, supervision, and supply chain management systems. Key informants in Malawi and Rwanda described these kinds of changes as happening after some level of iCCM institutionalization was perceived to be attained. Key informants in Mali described these efforts as happening even before institutionalization had been attained because most viewed institutionalization as only achieved when the decree formalizing the status of ASCs as recognized health workers was signed in 2022.

*I remember when we started working with [a particular NGO on supply chains], then as lessons came in, it was devolved to become a national system. There is a process to do quantification and then to procure to distribute to the districts. That one [supply chain system] continued developing until now, they have what they call the push and pull mechanism…CHWs don’t come to pick drugs, to pick supplies here [central level]. [Supplies] go through from central level. It goes to the district pharmacy, it goes to the hospital, it goes the health center. And then community health workers pick from the cell coordinators.* —UN key informant, Rwanda

In all 3 countries, key informants described continued adaptations to the service package provided by CHWs. In Malawi, additional services continued to be added to the HSA service package due to their recognition as a successful implementation platform. In Rwanda, the binôme service package also changed over time (e.g., addition of treatment of malaria in adults in 2013), although the most substantial change in CHW service packages was currently underway with the decision to adopt the polyvalent (i.e., multitask) CHW model in 2022. In Mali, key informants described that after the initial success of the program, other disease-specific health programs wanted to integrate services into the ASC package.

In all 3 countries, key informants described continued adaptations to the service package provided by CHWs.

*It’s good now, everybody will tell you iCCM is a huge contributor to the reduction of child mortality in Malawi. And that’s why you are seeing additional programs coming in, nutrition coming in, child protection coming in, all these other programs coming in [adding to the iCCM service package].* —UN key informant, Malawi

*Every program, HIV program, TB program, nutrition program etc. wanted to put its package in the ASC package because they figured they’re closer to the community and they’re the solution to our issues.* —NGO key informant, Mali

#### Maturity

Most key informants across all 3 countries expressed that institutionalization of iCCM had been “fully” achieved within their countries. This achievement was not described as guaranteeing the delivery of iCCM services perpetually but rather making it likely that iCCM services would continue while still being influenced by wider health system concerns and dynamics. Thus, key informants’ descriptions of institutionalization aligned with the iCCM Institutionalization Framework’s concept of the state of maturity in 2 significant ways. First, the majority of key informants described institutionalization as something that is achieved at some point and continues in the long term (i.e., it is a state that is stable). Second, several key informants described the importance of continually “strengthening” or reinforcing institutionalization. In other words, even if “full” institutionalization has been achieved, key informants still described ways that it could be strengthened or better sustained. In particular, key informants emphasized the need to secure sufficient financing and increases in domestic funds not only for iCCM services but also for the national health system as a whole. Several key informants highlighted that the long-term viability of iCCM institutionalization was inherently linked with this wider health system concern.

### Contributions of Institutionalization Drivers

Key informants were asked if and how each driver of the iCCM Institutionalization Framework (i.e., core values, leadership, policy, and resourcing) contributed to the advancement of iCCM institutionalization within their country. Often, key informants had already described the role of multiple drivers within the description of their country’s progression from iCCM introduction to scale-up to institutionalization before being explicitly asked. All key informants were asked to reflect on if and how the driver contributed to iCCM institutionalization from their perspective of dynamics and events within their country context. These descriptions were analyzed for patterns across key informants within each country and compared for similarities and differences across the 3 countries. Overall, key informants affirmed that each of the 4 drivers played important roles in their country’s institutionalization journey.

#### Core Values

Key informants described iCCM as aligning with the values of saving lives, bringing benefits to the community level, and responding to community needs (e.g., addressing barriers to health care access and the major causes of child death). These values were described as important for garnering initial support for iCCM across all 3 countries. Additionally, a key informant in Rwanda noted that iCCM was viewed as fitting within the national concept of “homegrown solutions,” which resulted in more political support for iCCM. In Malawi and Rwanda, several key informants noted that the perceived success of the iCCM platform (i.e., CHWs delivering services to the community level) drove continued interest in and support for iCCM.

*So, there were early adopters, but then there were also those that probably resisted. But we thought that we would not stop, because our target was the life of the child and then we were of the opinion that we have to continue and the rest of the people come and join. … But now it’s nice that almost now 80% or so of the people have accepted it as an intervention and accepted it as something that is contributed to the reduction of child mortality.* —UN key informant, Malawi

*What I can say is that [the] aspect of “homegrown solution” [drove] institutionalization of the initiative and the way it was implemented countrywide. You know, it was implemented countrywide with support from the president, from other ministries beyond the MOH. There was a lot of collaboration, a lot of joint effort.* —UN key informant, Rwanda

#### Leadership

Key informants emphasized the importance of leadership, including from the highest level of the MOH and wider government (e.g., cabinet, president, prime minister), as well as the importance of leadership at lower levels (e.g., the role of health district leaders and community leaders). In all 3 countries, several key informants expressed the view that establishment of a dedicated unit was important for institutionalization of iCCM (i.e., community health desk in Rwanda, the IMCI unit in Malawi, and national SEC coordination committee in Mali). In relation to the influence of government leadership, key informants also described the importance of governance mechanisms across management levels to ensure delivery of services and accountability. In Rwanda, performance-based contracts involving different levels of service delivery administrators and providers were described as an important mechanism. In Mali, an informant also described how community leaders contributed to the advancement of SEC by pressuring the coordinating associations and government to support it when there was a shortage of funding and resources.

*When we introduce the iCCM concept we normally invite the district health management team as an entry point to a meeting in their district, but also engage with the district councils. … We discuss, inform them, but also tell them about the intentions of the MOH. … including what are their roles and our expectations towards the implementation support towards iCCM in the district, so that led to easy implementation because we picked that as a format for introduction and scaling up of iCCM in all the districts in Malawi.* —Government key informant, Malawi

*And then you know, performance-based contracts are between the high-level leadership with the ministries, with the district, with the service providers […] down in the community. So, this one now ensures accountability, but also responsibility in terms of resources, in terms of people being responsible to what they are supposed to do.* —Government key informant, Rwanda

*Well, as I was saying in Mali, the advantage was that there was already an SEC focal point in the Ministry working with all of us. So that was already good. But it has to be said that the communities too, the community leaders who had seen this importance had contributed a great deal to ensuring that…they knew that if there was no ASC afterwards, it was going to be complicated. And so the local leaders continue to put the pressure on, even when there’s no money, to see how we can solve this problem.* —Government key informant, Mali

#### Policy

Key informants described policy as being essential to advancing institutionalization in that policy provides guidance and direction. Even more frequently than providing overall guidance, key informants described policy as evidence of government ownership and institutionalization itself. Across all 3 countries, iCCM strategies were developed and employed before becoming enshrined in relevant national policies and supported by decrees and laws. The timeline and types of policy documents that supported iCCM institutionalization varied across countries, influenced by the policy frameworks and mechanisms of each country. In some cases, policies and laws not directly referencing iCCM were deemed as ultimately important to advancing iCCM institutionalization. In Rwanda, the government’s decentralization policy (established in 2000) was referenced by a key informant as being important for underpinning and driving iCCM institutionalization. Finally, most key informants in Mali pointed to the presidential decree signed in 2021 making ASCs an official part of the health system as the penultimate indication of SEC institutionalization.

Key informants described policy as being essential to advancing institutionalization in that policy provides direction and demonstrates government ownership.

*Essentially at each and every level, wherever you are, there is no way you can implement something without a policy. So the policy acts as an enabler, it acts as a guide on what you are supposed to. So the policy is there. That’s why we have iCCM. So, whatever we are doing, we are guided by that IMCI or iCCM policy.*—Government key informant, Malawi

*Yes, I think this is what I said it’s institutionalized because we have a policy for [the] community health system. We have a strategic plan. We have even a ministerial instruction putting in place that system. So it is really in the institution. It is part of the health system.* —UN key informant, Rwanda

*I say it’s institutionalized now with the signing of the decree, it’s a presidential decree. —*Government key informant, Mali

#### Resources

Key informants across all countries described sufficient resources to be the most crucial for advancing iCCM institutionalization. Technical and financial support from external donors was described as playing a significant role in initiating the iCCM approach. Key informants described a need for additional human resources and supplies while scaling up iCCM services, but later, issues of maintaining a motivated workforce with sufficient pay and functional supply chains were a greater focus, including among CHWs interviewed. In Malawi, lack of sufficient financing for supplies and training was commonly highlighted as the key challenge for further expansion and institutionalization of iCCM. In Rwanda, multiple key informants noted the importance of increasing domestic financing to the health sector budget for sustaining the national health system, not only the delivery of iCCM services. In Mali, key informants emphasized the need to secure domestic financing (i.e., a budget line) for ASCs as key to strengthening and sustaining SEC institutionalization. Thus, narrative descriptions by key informants often focused on how lack of sufficient resources hinders or weakens iCCM institutionalization. While continued donor support was noted as important, several key informants described the importance of increasing domestic financing.

*I would simply say we are lacking support, especially for the expansion because we still have some hard-to-reach areas where iCCM hasn’t yet been reached. And the only limiting factor to that is that resources for training and resources for commodities and some equipment.* —Government key informant, Malawi

*I think if we look at the sustainability of even all the health gains achieved in Rwanda, I think the financing is one of the greatest challenges…I think it’s now about 40%–45% of the financing of the health sector is still done by the external. And even if you don’t look at the community level, I think already it gives you a picture of the dependency that we have vis*-à-*vis external financing.* —UN key informant, Rwanda

*As far as financial resources are concerned, I’d say it’s the fact that we don’t pay the ASCs. That’s the theme for the future [of institutionalization].* —Civil society key informant, Mali

## DISCUSSION

In recent years, several analytical reviews of existing literature and data have sought to identify factors influencing the implementation, scale-up, and institutionalization of iCCM.^15^^,^^27^^–^^29^ This study contributes to this expanding knowledge base by examining the perspectives of health system actors across 3 countries to understand their views on the definitions and processes of iCCM institutionalization. This approach allowed us to identify events and factors they viewed as significant within their national health systems over time through retrospective reflections.

This study contributes to this expanding knowledge base by examining the perspectives of health system actors across 3 countries to understand their views on the definitions and processes of iCCM institutionalization.

Key informants viewed government ownership and integration within national systems (i.e., iCCM services delivered through government-recognized health workers and supported through national supply chains, health information, and financing strategy) to define the status of iCCM institutionalization. While most emphasized the importance of aiming for national scale, they did not commonly use metrics of scale or coverage to describe the extent or status of institutionalization. A review of recent government documentation suggests that only Rwanda has achieved high levels of national scale of its community health program, with a total of 58,567 CHWs in 2021, aligning with the program’s design of 1 CHW per 200 persons.^30^ In contrast, Malawi’s community health program reached an estimated 50% of the population in 2021, with 8,778 HSAs, of whom 4,160 provide iCCM services in hard-to-reach areas.^31^ Meanwhile, Mali’s program was estimated to reach 23% of those living over 5 km from a health facility with 3,303 ASCs in 2021.^25^

The perception of iCCM as an integral part of a broader national health agenda rather than a separate initiative appears to have supported a sense of ownership, enabled health system integration, and advanced institutionalization from the perspective of key informants. This finding aligns with the WHO global review of IMCI, which discourages standalone approaches to iCCM in favor of those that are part of a systemwide strategy.^32^ In Mali, iCCM was not delineated by key informants from the country’s SEC package of services, so the introduction of iCCM was simply described as a component of the wider SEC package of services. Earlier research on iCCM adoption in Mali suggests MOH stakeholder concerns about the skill of existing volunteer CHWs (i.e., relais communautaires) to deliver iCCM services prompted the MOH to develop a new category of professionalized CHW (i.e., ASCs) to deliver the country’s SEC strategy, although this precipitating event was not mentioned by key informants in this study.^33^^–^^35^ In Rwanda, iCCM was described consistently as a component of the country’s overall community health strategy, regarded as having origins within the country’s community health program even before iCCM was piloted. In Malawi, iCCM appears to have had more delineation as an intervention, but it was viewed by key informants as building on the earlier IMCI program while also being described as a key feature of the country’s community health program. Previous research attributes Malawi’s early adoption of iCCM in part to how it built on the country’s previous IMCI program and was compatible with existing health infrastructure, including the prior existence of HSAs.^33^^,^^34^^,^^36^ Similarly, Rwanda had an existing community health program, which was leveraged to pilot iCCM with partner support and rapidly scale up iCCM services across the country.^37^ Despite variations in how existing CHWs and community health programs were adapted or leveraged to adopt iCCM, key informants across all 3 countries perceived iCCM as embedded within broader national health agendas.

While the iCCM institutionalization journeys described by key informants were unique to each country, all appeared to progress through the maturity phases as outlined in the iCCM Institutionalization Framework, although not as a linear journey. Key informants across all 3 countries depicted very rapid transitions from awareness and experimentation to expansion (less than 3 years), which were then followed by iterative phases of experimentation and consolidation over a period of at least 10 years. Several key informants emphasized the need to continue “strengthening” or reinforcing institutionalization even after it has been achieved so that it could be sustained within the context of wider health system dynamics. Sarriot and Kouletio have similarly theorized sustainability as a system struggling for equilibrium rather than a state to be reached.^38^ These findings align with the recent critical interpretive synthesis conducted by Kuchenmülle et al. on domains and processes for institutionalizing Evidence-Informed Policy, which found Evidence-Informed Policy institutionalization to be achieved through overlapping phases, iterative processes and the need to continually maintain or strengthen institutionalization.^39^

Key informants described the iCCM Institutionalization Framework’s drivers of core values, leadership, policy, and resourcing as contributing to the advancement of iCCM institutionalization within their countries. Overall, the influence of core values was described as stimulating initial interest and support for iCCM, which was then taken forward from experimentation to expansion by leadership within the MOH. Once within an expansion phase, the importance of policy was more frequently emphasized as needed to both guide strategic direction and demonstrate government ownership. Resources were commonly cited as the most important driver of institutionalization across all phases, starting with initial availability of sufficient human resources and supplies to pilot and scale-up but later shifting toward need for both sufficient long-term financing and domestic resources—an unfinished agenda in all 3 countries. Today, Malawi’s iCCM Investment Case (2021–2026) and Mali’s National Strategic Plan for Essential Care in the Community (2021–2025) present ambitious scale-up plans, reflecting additional iterative phases of expansion that require increased human resources and substantial financing.^25^^,^^31^ Malawi’s investment case projects financing gaps for the community health program to rise from US$5.4 million in 2021 to US$158.6 million in 2026.^31^ Mali’s strategic plan notes the importance of securing financing from the state budget, local authorities, communities, and NGOs, as well as technical and financial partners, but without estimating financing gaps.^25^ In contrast, Rwanda’s Community Health Investment Case estimates a much smaller annual financing gap of US$16 million from 2020 to 2030 to maintain established CHW structures and service delivery but notes a high level of donor dependence (i.e., an estimated 91% of the community health budget) and need for increases in government budgetary allocations.^30^ On the whole, the health institutions and systems of all 3 countries are highly dependent on development assistance for health.^40^

Overall, interviews with key informants across the 3 study countries allowed us to identify events and factors they considered significant for advancing iCCM institutionalization within their national health systems over time. These insights are valuable as they are informed by the contextual expertise and longterm perspectives of health system actors in each country, but multiple analytical approaches are needed to fully examine the complex dynamics of institutionalization. Future research could combine systematic document review, prospective analysis, and complex adaptive systems approaches with consideration of service delivery, health outcome, and financing metrics to further enrich understanding of institutionalization processes within national health systems.^29^^,^^41^^–^^43^

### Limitations

This study aimed to understand the iCCM institutionalization processes within the 3 selected study countries based on the perspectives of key informants engaged in iCCM within each country over a 20-year timeframe. It was not possible to obtain interviews with the full spectrum of individuals who could provide insight into all aspects of these processes. Notably, it was difficult to obtain interviews with individuals who were engaged in the earlier periods of iCCM introduction, particularly those who were retired and no longer associated with an organization in a professional capacity. Additionally, key informants often found it difficult to recall the details of events that happened many years prior. These challenges limited the information that could be gathered and analyzed from a firsthand perspective during the earlier periods of iCCM introduction, scale-up, and institutionalization.

Furthermore, resource constraints made it necessary to limit the total number of key informants who could be interviewed, and most interviews had to be conducted remotely. Scheduling interviews with identified potential key informants proved more challenging and time intensive than anticipated, including many instances of rescheduling interviews multiple times over several weeks. Conducting interviews remotely also presented challenges, including the absence of visual cues, connectivity problems, and difficulty reaching some individuals, which likely impacted the quality of the interviews. Finally, a few individuals identified as potential key informants declined to be interviewed, and some made it known they were not comfortable providing their personal viewpoints on the subject matter of the study. Altogether, these constraints likely limited the comprehensiveness and diversity of viewpoints shared by key informants and, thus, the richness of data in this qualitative study.

## CONCLUSION

This study has contributed to documenting and understanding the processes of iCCM institutionalization within Malawi, Mali, and Rwanda from the perspectives of health system actors. Overall, key informants viewed government ownership and integration within national systems (i.e., delivered through government-recognized health workers and supported through national supply chains, health information systems, and financing strategy) to define the status of iCCM institutionalization. While the iCCM institutionalization journeys of each country were unique, processes of institutionalization reflected a progression of maturity phases, which were iterative rather than linear in progression. Key informants emphasized the need to continually strengthen or reinforce iCCM institutionalization for it to be sustained within the context of wider health system dynamics. Future research involving systematic document review, prospective analysis, and complex adaptive systems approaches would further enrich understanding of how institutionalization occurs within national health systems. Further development of the iCCM Institutionalization Framework and other practical sensemaking models could assist health system actors in prioritizing their efforts to advance institutionalization of iCCM and other health interventions.

## Supplementary Material

GHSP-D-23-00509-Supplement.pdf

## Notes

**Peer Reviewed**

**First Published Online:** December 5, 2024.
