# Supplementary material for: Understanding Integrated Community Case Management Institutionalization Processes Within National Health Systems in Malawi, Mali, and Rwanda: A Qualitative Study
Source: Glob Health Sci Pract. 2024 Dec 20;12(6):e2300509. doi: 10.9745/GHSP-D-23-00509 (PMC11666091; doi:10.9745/GHSP-D-23-00509)
Supplement: GHSP-D-23-00509-Supplement.pdf [file GHSP-D-23-00509-Supplement.pdf]

Supplement to: Davis AL, Felker-Kantor E, Ahmed J, et al. Understanding integrated community case management institutionalization processes within national health systems in Malawi, Mali, and Rwanda: a qualitative study. *Glob Health Sci Pract*. 2024;12(6):e2300509. https://doi.org/10.9745/GHSP-D-23-000509

SUPPLEMENT 1. Semi-Structured Key Informant Interview Guide

1. What has been your engagement in [name of country]'s implementation of iCCM? Please mention the years of your engagement.

2. How would you describe [name of country]'s journey from introduction to nationwide scale of iCCM? What do you see as the major milestones? What factors do you think facilitated progress?

3. What challenges were encountered along this journey from introduction to nationwide scale of iCCM? How were these overcome? Who was involved and how? [Who can include particular groups of people (e.g. Ministry departments; donor agencies) and/or particular individuals]

4. From your perspective, to what extend is iCCM institutionalized into [name of country]'s health system? What do you see as evidence of iCCM institutionalization in [name of country]? How would you describe the status of national ownership of iCCM or community health more broadly? How would you describe the status of community human resources (i.e. CHWs) with the health system?

5. How would you describe [name of country]'s journey towards iCCM institutionalization? What do you see as the major milestones? What factors do you think facilitated progress? How do these compare or relate to the milestones and factors of scale-up?

6. What challenges have been encountered along this journey towards iCCM institutionalization? How have these been addressed and possibly overcome? Who was involved and how? [Who can include particular groups of people (e.g. Ministry departments; donor agencies) and/or particular individuals]

7. Do you think any particular beliefs or core values have motivated actors to support scale-up and institutionalization of iCCM in [name of country]? How were these expressed or demonstrated by actors involved in scale-up and institutionalization of iCCM? Did these beliefs or core values change overtime? If so, how?

8. What role has leadership played in [name of country]'s journey toward nationwide scale-up and institutionalization of iCCM? How have leaders within the public health system and government more broadly contributed to and/or hindered iCCM scale-up and institutionalization over the course of this journey?

9. How has health policy contributed to and/or hindered iCCM scale-up and institutionalization over the course of this journey? What challenges have been encountered? How have these been addressed? Who was involved and how? [Who can include particular groups of people (e.g. Ministry departments; donor agencies) and/or particular individuals]

10. How has the state of the health financing environment contributed to and/or hindered iCCM scale-up and institutionalization over the course of this journey? What challenges were experienced with financial, human resources or supplies? How have these been addressed and possibly overcome? Who was involved and how? [Who can include particular groups of people (e.g. Ministry departments; donor agencies) and/or particular individuals]

11. Reflecting back on [name of country]'s journey towards iCCM scale-up and institutionalization, what lessons learned or advice do you think would be useful to share with stakeholders in other countries? What lessons learned or advice do you think would be useful to share with global actors (e.g. UN technical agencies or donors)?

12. Is there anyone else that you would recommend that the research team speak with to better understand [name of country]'s journey towards iCCM scale-up and institutionalization? Would you be able and comfortable to share any contact details?

<u>Adjusted guide for community health workers only</u>

1. How long have you been a community health worker?

2. What services do you deliver as a community health worker?

3. Are you familiar with integrated community case management of childhood illness (iCCM)? How would you explain what it is?

4. Are you involved in providing iCCM services? If so, how?

5. How much time do you spend providing iCCM services? Has this changed over the time you have been a CHW?

5. Do members of your community know that you provide these kinds of services?

6. Do some people go elsewhere for these services? If so, where? Why?

7. Have you ever faced challenges in providing iCCM services? If so, what kinds of challenges?

8. Have the challenges you have faced changed over time? If so, how?

Supplement to: Davis AL, Felker-Kantor E, Ahmed J, et al. Understanding integrated community case management institutionalization processes within national health systems in Malawi, Mali, and Rwanda: a qualitative study. *Glob Health Sci Pract*. 2024;12(6):e2300509. https://doi.org/10.9745/GHSP-D-23-000509

Guide d'entretien semi-structuré avec les informateurs clés

1. Quel a été votre engagement dans la mise en œuvre des PCIME-C/SEC par [nom du pays]? Veuillez mentionner les années de votre engagement.

2. Comment décririez-vous le parcours de [nom du pays] de l'introduction à l'échelle nationale de la stratégie des PCIME-C/SEC? Quelles sont, selon vous, les principales étapes? Quels facteurs ont, selon vous, facilité les progrès?

3. Quels défis ont été rencontrés tout au long de ce parcours, de l'introduction à l'échelle nationale des PCIME-C/SEC? Comment ont-ils été surmontés ? Qui a été impliqué et comment? [Qui peut inclure des groupes particuliers de personnes (par exemple, des départements ministériels, des organismes donateurs) et/ou des individus particuliers].

4. De votre point de vue, dans quelle mesure la stratégie des PCIME-C/SEC est-elle institutionnalisée dans le système de santé de [nom du pays] ? Qu'est-ce que vous considérez comme une preuve de l'institutionnalisation des PCIME-C/SEC au [nom du pays]? Comment décririez-vous le statut de l'appropriation nationale des PCIME-C/SEC ou de la santé communautaire de manière plus générale? Comment décririez-vous le statut des ressources humaines communautaires (c.-à-d. les ASC) dans le système de santé?

5. Comment décririez-vous le parcours de [nom du pays] vers l'institutionnalisation des PCIME-C/SEC? Quelles sont, selon vous, les principales étapes ? Quels sont les facteurs qui, selon vous, ont facilité les progrès? Comment ces éléments se comparent-ils ou se rapportent-ils aux étapes et aux facteurs de la mise à l'échelle?

6. Quels défis ont été rencontrés au cours de ce voyage vers l'institutionnalisation des PCIME-C/SEC? Comment ont-ils été abordés et éventuellement surmontés? Qui a été impliqué et comment? [Qui peut inclure des groupes particuliers de personnes (par exemple, des départements ministériels, des organismes donateurs) et/ou des individus particuliers].

7. Pensez-vous que des croyances ou des valeurs fondamentales particulières ont motivé les acteurs à soutenir l'intensification et l'institutionnalisation des PCIME-C/SEC au [nom du pays]? Comment ont-elles été exprimées ou démontrées par les acteurs impliqués dans l'intensification et l'institutionnalisation des PCIME-C/SEC? Ces croyances ou valeurs fondamentales ont-elles évolué au fil du temps ? Si oui, comment?

8. Quel rôle le leadership a-t-il joué dans le parcours de [nom du pays] vers l'extension et l'institutionnalisation des PCIME-C/SEC à l'échelle nationale? Comment les dirigeants du système de santé publique et du gouvernement en général ont-ils contribué et/ou entravé l'intensification et l'institutionnalisation des PCIME-C/SEC au cours de ce parcours?

9. Comment la politique de santé a-t-elle contribué et/ou entravé l'extension et l'institutionnalisation des PCIME-C/SEC au cours de ce voyage? Quelles difficultés ont été rencontrées ? Comment ont-ils été relevés ? Qui a été impliqué et comment? [Qui peut

inclure des groupes particuliers de personnes (par exemple, des départements ministériels, des organismes donateurs) et/ou des individus particuliers].

10. Comment l'état de l'environnement financier de la santé a-t-il contribué et/ou entravé l'extension et l'institutionnalisation des PCIME-C/SEC au cours de ce voyage? Quelles difficultés ont été rencontrées en matière de ressources financières, humaines ou de fournitures? Comment ont-ils été abordés et éventuellement surmontés? Qui a été impliqué et comment? [Qui peut inclure des groupes particuliers de personnes (par exemple, des départements ministériels, des organismes donateurs) et/ou des individus particuliers].

11. En réfléchissant au parcours de [nom du pays] vers la mise à l'échelle et l'institutionnalisation des PCIME-C/SEC, quels enseignements ou conseils pensez-vous qu'il serait utile de partager avec les parties prenantes d'autres pays? Quels enseignements ou conseils pensez-vous qu'il serait utile de partager avec les acteurs mondiaux (par exemple, les agences techniques ou les donateurs des Nations Unies)?

12. Y a-t-il quelqu'un d'autre avec qui vous recommanderiez à l'équipe de recherche de s'entretenir pour mieux comprendre le parcours de [nom du pays] vers l'intensification et l'institutionnalisation des PCIME-C/SEC? Seriez-vous en mesure et à l'aise pour partager vos coordonnées?

Guide d'ajustement pour les agents de santé communautaires uniquement

1. Depuis combien de temps êtes-vous un agent de santé communautaire ?

2. Quels services fournissez-vous en tant qu'agent de santé communautaire ?

3. Connaissez-vous la gestion communautaire intégrée des maladies infantiles ? Comment expliqueriez-vous ce que c'est ?

4. Combien de temps passez vous à fournir des services PCIME-C/SEC? Cela a-t-il changé depuis que vous êtes ASC ?

5. Les membres de votre communauté savent-ils que vous fournissez ce type de services ?

6. Certaines personnes vont-elles ailleurs pour obtenir ces services ? Si oui, où ? Pourquoi ?

7. Avez-vous déjà été confronté à des défis dans la prestation des services PCIME-C/SEC? Si oui, quels types de défis ?

8. Les défis auxquels vous avez été confronté ont-ils changé avec le temps ? Si oui, comment ?

Supplement to: Davis AL, Felker-Kantor E, Ahmed J, et al. Understanding integrated community case management institutionalization processes within national health systems in Malawi, Mali, and Rwanda: a qualitative study. *Glob Health Sci Pract*. 2024;12(6):e2300509. https://doi.org/10.9745/GHSP-D-23-000509

SUPPLEMENT 2. DEDOOSE EXPORT OF CODEBOOK

TITLE	DESCRIPTION
**INFORMANT BACKGROUND & DEFINITIONS**	
Informant's Background	Informant's professional backgrounnd and experience working with iCCM.
Informant's Definition of iCCM	Key informant's description of what iCCM means to them within the context of
Informant's Definition of Institutionalization	Key informant's description of institutionalization, generally or in relation to iCCM specifically.
Informant's Description of iCCM Status	Key informant's description of the status of iCCM institutionalization in their country.
**INSTITUTIONALIZATION PROCESSES**	
Key Actors	Any mention of individuals or groups who have played an influential role in advancing or hindering iCCM institutionalization within the country.
Key Challenges	Any mention of challenges described as significant barriers or inhibitors to advancing iCCM institutionalization within the country.
Key Facilitators	Any mention of factors described as enabling iCCM institutionalization within the country.
Key Milestones/Turning Points	Any mention of particular events described as significant milestones or turning points in the country's journey towards iCCM institutionalization.
Key Strategies	Any mention of particular approaches used by an individual stakeholder or group of stakeholders to intentionally advance iCCM institutionalization.
**INSTITUTIONALIZATION FRAMEWORK DRIVERS**	
Core Values	Any mention of how beliefs or values have supported or hindered acceptance or support for iCCM.
Leadership	Any mention of how leaders individually or collectively supported or hindered advancing iCCM institutionalization, including those in formal or informal roles (e.g. those in senior government/ministry positions or other persons described as having significant influence across stakeholders).
Policy	Any mention of how policies or policy processes have supported or hindered advancing iCCM institutionalization.
Resources	Any mention of how the availability or lack of financial, human or material resources have supported or hindered advancing iCCM institutionalization.
**INSTITUTIONALIZATION FRAMEWORK PHASES**	
Awareness	Any mention of country stakeholders becoming aware of the need to improve management of childhood illness and iCCM as a possible approach.
Experimentation	Any mention of piloting or experimentating with some aspect of iCCM implementation in the country.
Expansion	Any mention of the expansion of iCCM implementation in the the country, including implementation across larger geographic areas, increases in population coverage or total number of cases treated.
Consolidation	Any mention of strengthening iCCM implementation to become a more routine health service delivery activity or more integral to the health system's service delivery model.
Maturity	Any mention of a state where iCCM is an integral, routine or stable part of the health system.
